# Real-Life Results of Palliative Chemotherapy in Metastatic Pancreatic Ductal Adenocarcinoma

**DOI:** 10.3390/cancers15133500

**Published:** 2023-07-05

**Authors:** Bianca Varzaru, Razvan A. Iacob, Adina E. Croitoru, Speranta M. Iacob, Cristina E. Radu, Stefania M. Dumitrescu, Cristian Gheorghe

**Affiliations:** 1 Faculty of Medicine, Carol Davila University of Medicine and Pharmacy, 020021 Bucharest, Romania; bianca.stoica@drd.umfcd.ro (B.V.); drcgheorghe@gmail.com (C.G.); 2 Gastroenterology Department, Sanador Clinical Hospital, 010991 Bucharest, Romania; 3 Digestive Diseases and Liver Transplantation Center, Fundeni Clinical Institute, 022238 Bucharest, Romania; 4 Oncology Department, Fundeni Clinical Institute, 022238 Bucharest, Romania; 5 Faculty of Medicine, Titu Maiorescu University, 040441 Bucharest, Romania; 6 Saint Mary Clinical Hospital, 011172 Bucharest, Romania

**Keywords:** metastatic pancreatic adenocarcinoma, palliative chemotherapy, overall survival, progression-free survival

## Abstract

**Simple Summary:**

The available chemotherapeutic regimens for metastatic pancreatic ductal adenocarcinoma (mPDAC) in Romania include FOLFIRINOX (FFX), gemcitabine-based regimens (GB), and gemcitabine monotherapy (Gem). The purpose of our study was to compare the efficacy of FFX, GB, and Gem in patients with mPDAC in real-world scenarios. Our research revealed that the overall survival (OS) of patients receiving FFX or GB was comparable, twice as long as that of patients receiving Gem, and the progression-free survival (PFS) was highest for patients receiving FFX as first-line chemotherapy (L1). Male gender, Eastern Cooperative Oncology Group Performance (ECOG-PS) 0/1, the FFX regimen, and neutrophil-to-lymphocyte ratio (NLR) > 4.15 were all independently linked to a longer OS. L1 with FFX improved both OS and PFS for second-line chemotherapy (L2).

**Abstract:**

Purpose: To assess the efficacy of FOLFIRINOX(FFX), gemcitabine-based regimens (GB), and gemcitabine monotherapy (Gem) in patients with metastatic pancreatic ductal adenocarcinoma (mPDAC). Methods: This is a retrospective study that included 83 patients with mPDAC treated with first-line chemotherapy (L1) with either FFX, GB or Gem between 2015 and 2017. Progression-free survival (PFS) for L1 and second-line chemotherapy (L2) (PFS-L1 and PFS-L2) and overall survival (OS) were estimated using the Kaplan–Meier method. Results: Median PFS-L1 for FFX, GB and Gem groups was 9 months (95% (Confidence Interval) CI 2.76–15.24), 5 months (95%CI 3.44–6.56), and 5 months (95%CI 3.76–6.24), respectively (*p* = 0.04). OS was 14 months (95%CI 11.16–16.85), 12 months (95%CI: 9.44–11.56), and 7 months (95%CI: 5.7–8.3) for patients treated with FFX, GB, and Gem, respectively (*p* = 0.0001). ECOG-PS (0/1) (Hazard Ratio (HR) 6.74, *p* = 0.002), age > 70 years (HR 0.25, *p* = 0.04), body tumors (HR 2.8, *p* = 0.048), CA19–9 > 39 U/mL (HR 0.26, *p* = 0.02), and neutrophil-to-lymphocyte ratio (NLR) > 4.15 (HR 6.76, *p* = 0.001) were independent prognostic factors for PFS-L1. Male gender (HR 3.02, *p* = 0.026), ECOG-PS (0/1) (HR 4.21, *p* = 0.003), L1 with FFX (HR 0.255, *p* = 0.007), and NLR > 4.15 (HR 2.65, *p* = 0.04) were independent prognostic factors of OS. PFS-L2 (HR 6.91, *p* = 0.013) and OS-L2 (HR 6.95, *p* = 0.037) were significantly higher in patients first treated with FFX. Conclusions: The OS of patients who receive FFX or GB is comparable. The best PFS-L1 belongs to the FFX group. Male gender, ECOG-PS 0/1, the FFX regimen, and NLR > 4.15 were independent predictors of OS. PFS-L2 and OS-L2 were favorably impacted by L1 with FFX.

## 1. Introduction

Pancreatic adenocarcinoma is one of the most lethal cancers in the world, with an incidence rate approximately equal to the mortality rate [1,2]. Although diagnosis accuracy has greatly improved over the past two decades [3], 28% of patients are diagnosed with locally advanced pancreatic cancer and 53% with metastatic disease [4], with median OS of approximately 12 months and 6 months, respectively [5]. The optimal treatment for pancreatic cancer remains controversial. Resection is the only curative therapy for patients with localized pancreatic cancer; however, management is mainly limited to chemotherapy, sometimes combined with radiotherapy for loco-regional diseases [6].

Gem was the usual chemotherapy regimen until 2011, when FFX was presented as a novel, more efficient L1 regimen for advanced pancreatic cancer. In 2011, Conroy et al. (PRODIGE trial) showed that the median OS for chemotherapy-naive patients treated with FFX was approximately 11 months, compared to 6.8 months for patients treated with Gem, with median PFS of 6.4 months and 3.3 months, respectively (HR, 0.47, 95% CI 0.37 to 0.59; *p* = 0.001) [7]. Patients with mPDAC who received gemcitabine combined with nanoparticle albumin-bound (nab)-paclitaxel (nab-paclitaxel) had a significantly better OS (8.5 vs. 6.7 months) as compared to those who received Gem, according to Von Hoff et al. [8].

When recommending L2 to patients with mPDAC, the L1 regimen should be taken into account. [7,9]. FFX is often administered first when patients are in relatively good general condition [8]. Less than half of patients with advanced or mPDAC who experienced L1 treatment failure remain in good general condition and may receive subsequent L2 regimens [10]. Combinations of 5-fluorouracil (FU) with oxaliplatin [11] or nanoliposomal irinotecan [9] can be suggested as L2 after Gem fails.

Even though FFX and gemcitabine plus nab-paclitaxel are both considered standard L1 [7,8] for patients with PDAC, nab-paclitaxel is not currently covered by insurance in our country. At this moment, the palliative chemotherapy regimens available in our country are based on FFX, GB (gemcitabine combined with capecitabine, cisplatin, or oxaliplatin), or Gem. The choice of L1 takes into account the patient’s characteristics: age, ECOG-PS, bilirubin levels, nutritional status, and comorbidities.

The aim of this study was to compare the efficacy of the chemotherapy regimens available in our country (FFX, GB, and Gem) and identify the prognostic factors of OS and PFS in a real-world setting.

## 2. Materials and Methods

We analyzed data from 83 consecutive patients diagnosed with mPDAC between 2015–2017 in the Oncology Department of Fundeni Clinical Institute (Bucharest, Romania). In this research, only patients who received FFX, GB or Gem were considered. Pathological confirmation was provided for each patient. Since this group of patients was retrospectively reviewed, there were no ethical issues.

Medical record variables were gathered and entered into a database. Baseline characteristics (age, gender, tumor location, ECOG-PS, presence of diabetes mellitus, CA 19-9 levels), as well as NLR and L1 and L2 regimens, were analyzed with respect to their prognostic significance. OS was defined as the period from the time of diagnosis until death (from complications related to pancreatic cancer or other causes) or the end of follow-up. PFS-L1 was calculated from the first chemotherapy dosage to either the first sign of tumor progression or the patient’s death, whichever came first. For patients who received L2 therapy, the OS-L2 and PFS-L2 were computed. The OS-L2 was calculated starting with the date of progression under L1 (or the date of initial administration of L2 if no progression was observed under L1) and ending with the date of death from any cause. PFS-L2 was computed from the date of progression under L1 (or the date of first administration of L2 if no progression was recorded under L1) until the date of progression, death from any cause, or the end of follow-up. Survival data were censored at the last follow-up. The cut-off date for follow-up was 30 June 2019.

SPSS statistical software (IBM SPSS Statistics 20.0) was used for all analyses. According to the Kaplan–Meier method, a survival analysis was conducted, and survival curves were compared using the log rank test, considering *p* < 0.05 for statistical significance. The optimal cut-off value for baseline NLR was determined using a time-dependent receiver operating characteristic (ROC) analysis, considering 6-month survival as the reference time point.

The multivariate survival analysis was further conducted using the Cox regression model, submitting into analysis variables that were found to be significant (*p* < 0.05) in univariate analysis and adjusting for age and ECOG-PS.

The study’s goals were to assess the effectiveness of the chemotherapy regimens that are used in our nation and to pinpoint the variables that affect OS and PFS in the real world.

## 3. Results

### 3.1. Patients’ Characteristics

Between January 2015 and December 2017, 83 consecutive patients diagnosed with mPDAC and treated with chemotherapy regimens in the oncology department were included in this analysis. Clinical parameters, information on the L1 and L2 regimens, PFS, and OS were recorded.

Table 1 displays demographic information and disease features at the beginning of the L1 regimens. The male-to-female ratio was 48:35, and the median age was 63 years (interquartile range (IQR), 57–71). The median age of the patients in the Gem group was 67 years (IQR: 58.5–73), compared to 61 years (IQR: 54–66) for the GB group and 60 years (IQR: 54–65) for the FFX group. In about 54% of patients, the tumor was located in the head or uncinate process of the pancreas; in 27% of patients, it was found in the pancreatic corpus, and in 19% of patients, it was found in the tail of the pancreas. The patients in the analyzed group had an ECOG-PS of 0/1 or 2 (51.8% and 48.19%, respectively), and 37.4% of them suffered from type 2 diabetes. The majority of patients (almost 81%) had elevated baseline levels of CA 19-9 (>39 UI/mL), and the median NLR was 3.35 (IQR: 2.14–4.75). Gem was used as the L1 regimen in 53.01% of cases, GB in 24.09% of cases, and FFX in 22.9% of cases. Almost 29% of patients (*n* = 24) underwent L2 regimens. Patients treated with L2 regimens received 5-FU plus leucovorin, irinotecan, and/or oxaliplatin (*n* = 11, 45.83%), Gem (*n* = 5, 20.83%), GB (*n* = 5, 20.83%), and other chemotherapy, such as oxaliplatin, irinotecan, or capecitabine (*n* = 3, 12.5%).

### 3.2. Statistical Analysis

**Figure 1:**
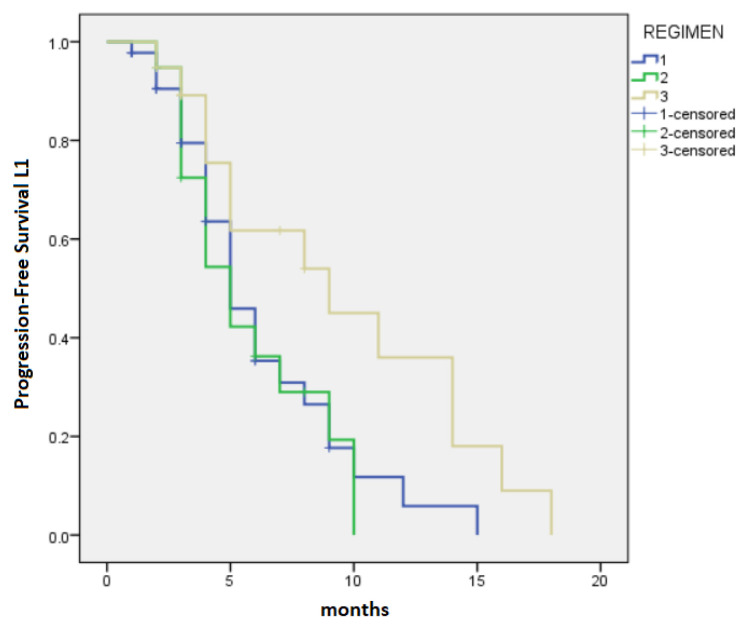
Median progression-free survival for first-line chemotherapy: 1—Gemcitabine monotherapy: 5 months, 2—Gemcitabine-based regimens: 5 months, 3—FOLFIRINOX: 9 months (*p* = 0.044).

**Figure 2:**
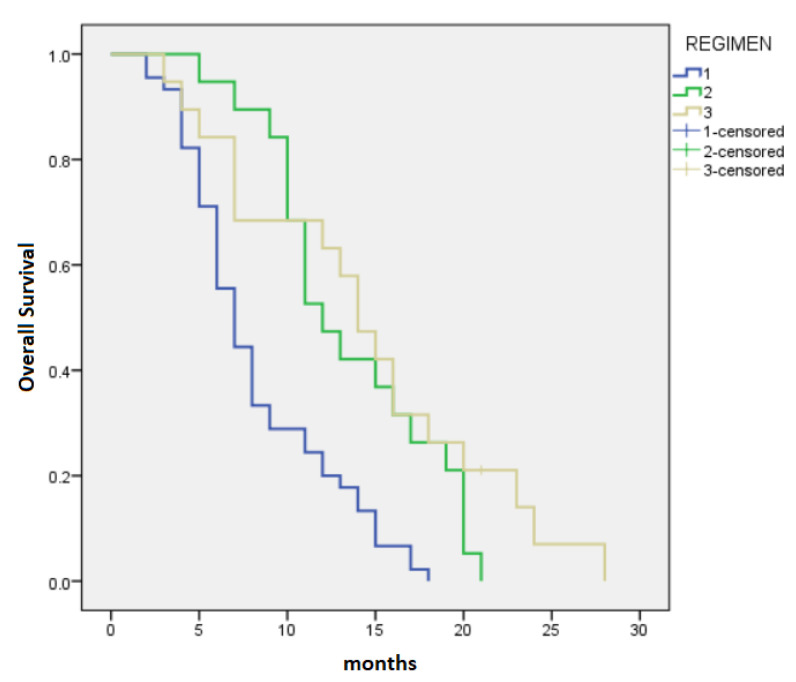
Median overall survival: 1—Gemcitabine: 7 months, 2—Gemcitabine-based regiments: 12 months 3—FOLFIRINOX: 14 months (*p* < 0.0001).

The median PFS-L1 and median OS were 5 months (95% CI 3.88–6.12) and 10 months (95% CI 7.77–12.23), respectively, with variation regarding the chemotherapy regimens. Patients in the FFX group had a median PFS-L1 of 9 months (95% CI 2.76–15.24), whereas the GB group and Gem group had median PFS-L1 of 5 months (95% CI 3.44–6.56) and 5 months (95% CI 3.76–6.24), respectively (*p* = 0.044) (Figure 1). OS for patients treated with FFX was 14 months (95% CI 11.16–16.85), for patients treated with GB, it was 12 months (95% CI 9.44–14.56), and for patients treated with Gem, it was 7 months (95% CI 5.7–8.3), *p* < 0.0001 (Figure 2).

At 6, 12, 18, and 24 months, the OS rates in the Gem group were 71.11%, 24.44%, 2.22%, and 0%, compared to 94.74%, 57.9%, 26.32%, and 0% in the GB group and 84.21%, 68.42%, 31.58%, and 10.52%, respectively, in the FFX group.

**Figure 3:**
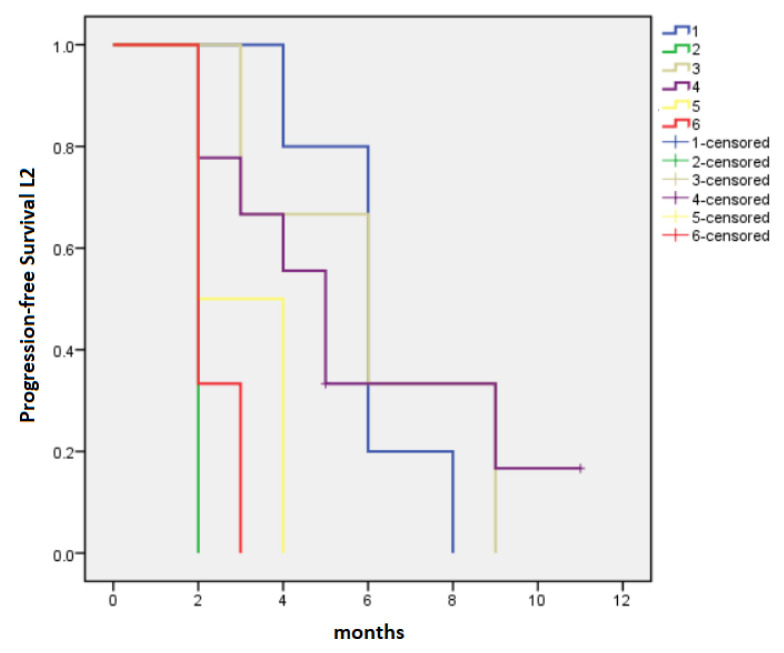
Median progression-free survival for second-line chemotherapy: 1—FOLFIRINOX → Gemcitabine monotherapy (6 months), 2—Gemcitabine monotherapy → 5-FU-based regimens (2 months), 3—FOLFIRINOX → Gemcitabine-based regimens (6 months), 4—Gemcitabine-based regimens → 5—FU-based regimens (5 months), 5—Gemcitabine monotherapy → Gemcitabine-based regimens (2 months), 6—others (2 months), *p* = 0.014.

**Figure 4:**
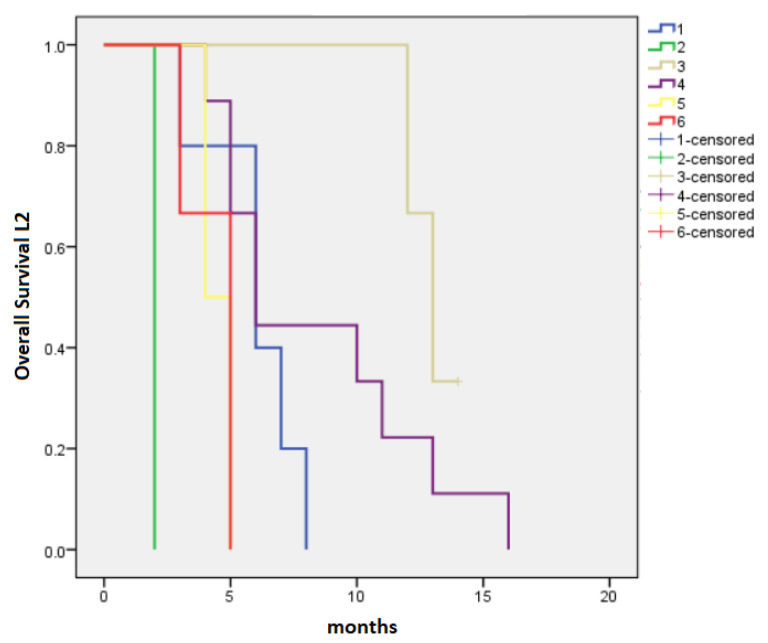
Median overall survival for second-line chemotherapy: 1—FOLFIRINOX → Gemcitabine monotherapy (6 months), 2—Gemcitabine monotherapy → 5-FU-based regimens (2 months), 3—FOLFIRINOX → Gemcitabine-based regimens (13 months), 4—Gemcitabine-based regimens → 5-FU-based regimens (6 months), 5—Gemcitabine monotherapy → Gemcitabine-based regimens (4 months), 6—others (5 months), *p* < 0.0001.

The median PFS-L2 and OS-L2 for patients who received L2 were 4 months (95% CI 2.16–5.84) and 6 months (95% CI 4.97–7.03), respectively. PFS-L2 was longer in patients initially treated with FFX followed by Gem (PFS-L2 = 6 months, 95% CI 4.83–7.17) or GB (PFS-L2 = 6 months, 95% CI 1.2–10.8), as well as in patients receiving in L1 GB followed in L2 by 5-FU-based regimens (PFS-L2 = 5 months, 95% CI 3.61–6.38), *p* = 0.014 (Figure 3). The longest OS-L2 was recorded in patients treated with the sequence FFX → GB (13 months, 95% CI 11.4–14.6), followed by those treated with FFX → Gem (6 months, 95% CI 2.77–9.22) and GB → 5-FU-based regimens (6 months, 95% CI 4.54–7.46), *p* < 0.0001 (Figure 4).

A Cox regression model was used to assess the relationship between patients’ characteristics and survival. This analysis took into account factors like age, gender, tumor location, diabetes, ECOG-PS, L1 and L2 regimens, CA 19-9, and NLR at diagnosis. A time-dependent receiver operating characteristic (ROC) analysis was conducted using 6-month survival as the reference time point in order to determine the cut-off value for baseline NLR. The optimal cut-off value for baseline NLR was 4.15, with an area under the curve (AUC) of 0.754 (95% CI 0.594–0.913, *p* = 0.003).

The multivariate survival analysis was further conducted using the Cox regression model. Multivariate survival analyses were performed by submitting into analysis variables that were found to be significant (*p* < 0.05) or near significant in univariate analysis and by adjusting for age and ECOG-PS.

In univariate analyses, CA 19-9 > 39 UI/mL (HR 2.46, 95% CI 1.18–5.14, *p* < 0.016), NLR > 4.15 (HR 0.52, 95% CI 0.29–0.92, *p* = 0.024), L1 with FFX vs. Gem (HR 2.42, 95% CI 1.06–5.49, *p* = 0.035), and L1 with GB vs. Gem (HR 2.13, 95% CI 1.03–4.39, *p* = 0.041) were prognostic factors of the PFS-L1. In the multivariate Cox regression model, age > 70 years (HR 0.25, 95% CI 0.07–0.95, *p* = 0.04), ECOG-PS (0/1 vs. 2) (HR 6.74, 95% CI 1.99–22.87, *p* = 0.002), tumor location (corpus vs. head) (HR 2.8, 95% CI 1.01–7.73, *p* = 0.048), increased CA 19-9 (>39 UI/mL) at diagnosis (HR 0.26, 95% CI 0.08–0.83, *p* = 0.02), and NLR > 4.15 at diagnosis (HR 6.76, 95% CI 2.24–20.37, *p* = 0.001) were all independent predictors of PFS-L1. ECOG-PS 0/1 was associated with a longer PFS-L1, while age > 70, elevated CA 19-9 levels, NLR > 4.15, and tumor in the pancreatic body were linked with a shorter PFS-L1 (Table 2). There was no statistically significant correlation between PFS-L1 and gender, diabetes, or chemotherapy treatment.

In univariate analysis, baseline ECOG-PS 0/1 vs. 2 (HR 2.49, 95% CI 1.56–3.98, *p* < 0.0001), NLR > 4.15 (HR 0.6, 95% CI 0.37–0.97, *p* = 0.036), L1 with FFX vs. Gem (HR 0.29, 95% CI 0.16–0.56, *p* < 0.0001), and L1 with GB vs. Gem (HR 0.41, 95% CI 0.23–0.72, *p* = 0.002) were independent prognostic factors of OS. In the multivariate analysis, male gender (HR 3.02, 95% CI 1.14–7.98, *p* = 0.026), ECOG-PS (0/1 vs. 2) (HR 4.21, 95% CI 1.61–11, *p* = 0.003), palliative chemotherapy FFX (HR 0.255, 95% CI 0.09–0.69, *p* = 0.007), and baseline NLR > 4.15 (HR 2.65, 95% CI 1.04–6.75, *p* = 0.04) were the independent prognostic factors of OS. Male gender, ECOG-PS 0/1, and L1 with FFX (vs. Gem) were linked to improved OS, whereas higher NLR was linked to lower OS. A worse OS was also indicated by increased CA 19-9 at diagnosis, although the association lacked statistical significance (HR 0.41, 95% CI 0.17–1, *p* = 0.05) (Table 3). In the multivariate analysis, L1 with FFX (vs. Gem) was an independent predictor for PFS-L2 (HR 6.91, 95% CI 1.5–31.9, *p* = 0.013) and OS-L2 (HR 6.95, 95% CI 1.12–43.07, *p* = 0.037). OS-L2 and PFS-L2 did not correlate statistically significantly with gender, age at the start of L2, or the L2 regimen.

## 4. Discussion

In this study, we retrospectively analyzed the clinical outcomes of patients with mPDAC. The patient characteristics included in our study were similar to those published in previous epidemiological studies in terms of gender, age, and tumor location [5,7,12,13]. Consistent with the results of previous studies [7,12], we noticed significant improvement in OS in patients treated with FFX or GB regimens [14] compared to patients receiving Gem (12 months and 14 months, respectively, vs. 8 months).

A retrospective multinational study, which included 1089 patients from the UK, Sweden, Germany, Italy, and Hungary with mPDAC undergoing palliative chemotherapy between 2012 and 2015, analyzed the efficacy of FFX and gemcitabine plus nab-paclitaxel versus Gem and GB regimens like gemcitabine plus oxaliplatin, gemcitabine plus cisplatin, and gemcitabine plus capecitabine. The median OS was 9 months for the FFX group (95% CI 7.1–10.9), 7 months for gemcitabine plus nab-paclitaxel (95% CI 5.8–8.2), 7 months for GB regimens (95% CI 6.0–8.0), and 5 months for the Gem group (95% CI 4.3–5.7) [15].

It is interesting to note that a recent systematic review comparing the safety and efficacy of FFX to gemcitabine plus nab-paclitaxel and gemcitabine plus capecitabine in the treatment of pancreatic cancer revealed that FFX was linked to significantly longer survival than gemcitabine plus nab-paclitaxel and gemcitabine plus capecitabine. In the adjuvant pancreatic cancer setting, combination regimens (FFX, gemcitabine plus capecitabine, and gemcitabine plus nab-paclitaxel) had statistically significant differences for OS (HR 0.78, 95% CI: 0.69–0.89) and relapse-free survival/disease-free survival (HR 0.77, 95% CI 0.60–0.98) compared to Gem. Also, in advanced/metastatic pancreatic cancer, FFX showed significantly better OS (HR 0.71, 95% CI 0.60–0.85) and PFS (HR 0.65, 95% CI 0.57–0.74) compared to gemcitabine plus capecitabine [16].

In our retrospective cohort, the proportion of patients receiving L2 was ~29%, which is consistent with reports from other studies [17,18]. A retrospective study on real-world unselected patients with pancreatic cancer reported a median OS-L2 of 5.2 months (95% CI 4.7–5.7) [17]. In another retrospective study on 366 patients with advanced pancreatic cancer who had received palliative chemotherapy, 28.4% underwent L2, the median OS-L2 was 6.4 months (95% CI 4.5–8.6), and the median PFS-L2 was 4.5 months (95%CI 2.7–6.3) [18]. Despite the lack of statistical significance, Gränsmark et al. noticed a better OS-L2 in patients receiving gemcitabine plus nab-paclitaxel as L1 compared to the Gem subgroup (HR = 0.70, 95% CI 0.48–1.03, *p* = 0.067) [17].

A Cox regression model was used to assess the relationship between patients’ characteristics and survival. In our cohort study, the multivariate analysis showed that ECOG-PS 0/1 was associated with prolonged PFS-L1, while age over 70, increased levels of CA 19-9, NLR > 4.15, and tumors located in the pancreatic body negatively influenced PFS-L1. Greater OS was linked to male gender, ECOG-PS 0/1, and the L1 regimen with FFX (vs. Gem), while lower survival was linked to higher NLR. A worse survival was also predicted by higher CA 19-9 levels at diagnosis, although this prediction was not statistically significant (HR 0.41, 95% CI 0.17–1, *p* = 0.05). Furthermore, L1 with FFX (vs. Gem) was an independent predictor for PFS-L2 (HR 6.91, 95% CI 1.5–31.9, *p* = 0.013) and OS-L2 (HR 6.95, 95% CI 1.12–43.07, *p* = 0.037).

Artinyan et al. examined the relationship between tumor location and survival. In multivariate analysis, body and tail locations were significant prognostic factors for decreased survival (HR 1.11, 95% CI 1.00–1.23, *p* = 0.05) [19]. Further, in a systematic review and meta-analysis of 93 studies, studying the outcome of head compared to body and tail pancreatic cancer, multivariate analysis showed that the head site was an independent prognostic factor for survival (HR 0.95, 95% CI 0.92–0.99, *p* = 0.02), and tumors of the head were associated with a similar PFS to tail cancers (HR 0.99, 95% CI 0.84–1.16, *p* = 0.91) [20]. In another study, normal baseline albumin (HR 0.63, 95% CI 0.41–0.97), male sex (HR 0.65, 95% CI 0.43–0.97), and L2 therapy (HR 0.50, 95% CI 0.28–0.86) were correlated with better survival [21].

In a multivariate analysis of 154 patients with inoperable pancreatic cancer treated with Gem and/or 5-FU-based therapy, the baseline CA 19-9 above or below the median value (958 U/mL) was an independent prognostic factor for OS (HR 1.8, 95% CI 1.3–2.5, *p* = 0.0004) [22]. In a retrospective analysis, patients with increased levels of the biomarkers CA 19-9 and CEA, or the location of cancer in the pancreatic tail, were associated with a worse prognosis.

Based on our cohort data and according to the ROC curve, the cut-off value of the NLR was 4.15, which is consistent with that in previous studies [23,24,25,26]. In several studies, multivariate analysis revealed that elevated NLR (cut-off values ranged from 2 to 5) indicated a worse prognosis than in patients with lower NLR [27,28].

A systematic review published in 2013 [29], analyzing the relationship between the NLR and cancer outcome (OS/cancer-specific survival/disease recurrence, or response to treatment), demonstrated that the NLR had prognostic value in a variety of tumor types, and in cancer patients receiving chemo/radiotherapy, elevated baseline NLR was associated with poor OS.

Several retrospective studies analyzing patients with locally advanced or metastatic pancreatic cancer treated with palliative chemotherapy assessed the prognostic value of NLR. For example, Luo et al. retrospectively reviewed 403 patients undergoing chemotherapy for advanced pancreatic adenocarcinoma and showed that both baseline NLR (NLR > 3.1, HR = 1.42; *p* = 0.001) and post-treatment NLR (NLR > 3.1, HR = 2.39; *p* > 0.0001) were independent prognostic factors of OS [25]. An et al. demonstrated that elevated pre-treatment NLR (> 5) was a predictor of shorter survival in patients with advanced pancreatic cancer (*p* = 0.001) [30]. Also, in another retrospective study, a multivariate analysis revealed that a high NLR > 3.74 (HR 2.430; 95% CI 1.484–3.977; *p* < 0.001) was independently associated with OS [24].

## 5. Conclusions

Oncologists continue to face difficulties in the management of mPDAC. In this study, FFX was an effective L1 option for patients with ECOG-PS 0/1.

The patients with the best PFS-L1 (9 months) in our real-life mPDAC cohort scenario are those who received FFX as an L1 regimen. Patients who underwent either the FFX or GB regimens had similar OS (14 months and 12 months, respectively).

Our results demonstrate that age over 70 years, increased levels of CA 19-9, NLR > 4.15, and tumors located in the pancreatic body negatively influenced PFS-L1, while ECOG-PS 0/1 was associated with prolonged PFS-L1. None of the following factors were statistically significantly linked with PFS-L1: gender, diabetes, or chemotherapy regimen. Male gender, ECOG-PS 0/1, and L1 with FFX (vs. Gem) were associated with better OS, while increased NLR was correlated with decreased OS. At the same time, patients with elevated CA 19-9 values at diagnosis had lower OS. In this study, around one-third of the patients received L2, and regardless of the L2 regimen, those who received FFX first had significantly greater PFS-L2 and OS-L2 than those who did not. There is very limited information available regarding the treatment strategies for pancreatic cancer in Eastern Europe. As far as we are aware, this is the first study comparing the effectiveness of mPDAC palliative chemotherapeutic options currently available in Romania. We believe that our research’s conclusions may be helpful in assisting oncologists in making decisions regarding their choice of chemotherapy regimens given the difficulties they experience in controlling mPDAC.

There are some limitations in this research, as it is a retrospective study performed in a single center with a small number of patients. Additionally, due to the study’s retrospective character, no information on the patients’ psychosocial or emotional effects could be gathered, despite the fact that an increasing amount of research suggests a connection between psychological stress and site-specific cancer mortality. To confirm our findings, we think a multicenter, nationwide prospective validation is required.

## Figures and Tables

**Table 1:** Baseline characteristics of patients treated with palliative chemotherapy.

Variable	All (*n* = 83)	Gem (*n* = 44)	GB (*n* = 20)	FFX (*n* = 19)
Age (years)	63 (57–71)	67 (58.5–73)	61 (54–66)	60 (54–65)
Age ≥ 70 years, *n* (%)	23 (27.71)	19 (43.18)	2 (10)	2 (10.53)
Sex, male, *n* (%)	48 (57.83)	20 (45.45)	13 (65)	14 (73.86)
ECOG-PS				
0/1, *n* (%)	43 (51.8)	14 (31.81)	12 (60)	17 (89.47)
2, *n* (%)	40 (48.19)	30 (68.18)	8 (40)	2 (10.53)
Primary tumor location				
head/uncinate process, n (%)	45 (54.22)	26 (59.09)	9 (45)	10 (52.63)
body, *n* (%)	22 (26.5)	12 (27.27)	5 (25)	5 (26.32)
tail, *n* (%)	16 (19.28)	6 (13.63)	6 (30)	4 (21.05)
Diabetes Mellitus, *n* (%)	31 (37.35)	18 (40.90)	4 (20)	9 (47.37)
CA 19-9				
>39 UI/mL	67 (80.72)	36 (81.82)	15 (75)	16 (84.21)
≤39 UI/mL	16 (19.28)	9 (18.18)	5 (25)	3 (15.79)
NLR	3.35 (2.14–4.52)	3.9 (2.55–4.75)	3.28 (2.17–3.95)	2.72 (1.61–4.19)

All variables are provided as median (IQR) or number (percentage). Abbreviations: Gem: Gemcitabine monotherapy, GB: gemcitabine-based regimens, FFX: FOLFIRINOX, ECOG-PS: Eastern Cooperative Oncology Group Performance Status, NLR: neutrophil-to-lymphocyte ratio.

**Table 2:** Prognostic factors of the progression-free survival for first-line chemotherapy.

Prognostic Factor	Univariate Analyses		Multivariate Analyses	
	*p*	HR (95% CI)	*p*	HR (95% CI)
Age (years)	0.68	0.99 (0.97–1.02)		
Age > 70 years	0.65	1.16 (0.62–2.14)	0.04	0.25 (0.07–0.95)
Sex: male vs. female	0.58	1.16 (0.67–2.02)	0.21	0.52 (0.18–1.44)
Diabetes	0.55	1.18 (0.68–2.05)		
Baseline ECOG-PS (0/1 vs. 2)	0.063	0.58 (0.33–1.03)	0.002	6.74 (1.99–22.87)
Tumor location				
head vs. body	0.22	2.02 (1.11–3.69)	0.048	2.8 (1.01–7.73)
head vs. tail	0.73	1.13 (0.54–2.37)	0.142	2.75 (0.71–10.6)
CA 19-9 > 39 UI/mL vs. ≤ 39 UI/mL	0.016	2.46 (1.18–5.14)	0.02	0.26 (0.08–0.83)
NLR > 4.15 vs. NLR ≤ 4.15	0.024	0.52 (0.29–0.92)	0.001	6.76 (2.24–20.37)
First-line chemotherapy				
FFX vs. Gem	0.035	2.42 (1.06–5.49)	0.37	0.47 (0.08–2.5)
GB vs. Gem	0.041	2.13 (1.03–4.39)	0.97	0.98 (0.32–2.96)

Abbreviations: ECOG-PS: Eastern Cooperative Oncology Group Performance Status, FFX: FOLFIRINOX, Gem: Gemcitabine monotherapy, GB: gemcitabine-based regimens, NLR: neutrophil-to-lymphocyte ratio.

**Table 3:** Prognostic factors of overall survival.

Prognostic Factor	Univariate Analyses		Multivariate Analyses	
	*p*	HR (95% CI)	*p*	HR (95% CI)
Age (years)	0.879	1.01 (0.98–1.02)		
Age > 70 years	0.279	0.76 (0.47–1.24)	0.33	0.62 (0.24–1.61)
Sex: male vs. female	0.685	1.1 (0.70–1.71)	0.026	3.02 (1.14–7.98)
Diabetes	0.571	1.14 (0.72–1.79)		
Baseline ECOG-PS (0/1 vs. 2)	0.0001	2.49 (1.56–3.98)	0.003	4.21 (1.61–11)
Tumor location				
head vs. body	0.881	1.04 (0.62–1.75)		
head vs. tail	0.756	0.91 (0.50–1.64)		
CA 19-9 > 39 UI/mL vs. ≤ 39 UI/mL	0.460	1.23 (0.71–2.14)	0.05	0.41 (0.17–1)
NLR > 4.15 vs. NLR ≤ 4.15	0.036	0.60 (0.37–0.97)	0.04	2.65 (1.04–6.75)
First-line chemotherapy				
FFX vs. Gem	0.0001	0.29 (0.16–0.56)	0.007	0.26 (0.09–0.69)
GB vs. Gem	0.002	0.41 (0.23–0.72)	0.253	0.49 (0.14–1.68)

Abbreviations: ECOG-PS: Eastern Cooperative Oncology Group Performance Status, FFX: FOLFIRINOX, Gem: Gemcitabine monotherapy, GB: gemcitabine-based regimens, NLR: neutrophil-to-lymphocyte ratio.

## Data Availability

Data are available upon reasonable request and with the permission of the corresponding author.
